# EnLightenment: High resolution smartphone microscopy as an educational and public engagement platform

**DOI:** 10.12688/wellcomeopenres.12841.2

**Published:** 2018-05-03

**Authors:** Laura C. Wicks, Gemma S. Cairns, Jacob Melnyk, Scott Bryce, Rory R. Duncan, Paul A. Dalgarno

**Affiliations:** 1 HW Engage, Heriot-Watt University, Edinburgh, EH14 4AS, UK; 2 Institute of Biological Chemistry, Biophysics and Bioengineering, Scottish Universities Physics Alliance (SUPA), Heriot-Watt University, Edinburgh, EH14 4AS, UK; 3 Centre for Doctoral Training in Medical Devices and Health Technologies, Technology and Innovation Centre, University of Strathclyde, Glasgow, G1 1XQ, UK; 4 West Calder High School, West Calder, EH55 8QN, UK; 5 Scottish Schools Educational Research Centre (SSERC), Dunfermline, KY11 8UU, UK

**Keywords:** Microscopy, Imaging, Smartphone, Public Engagement, Education, Resolution, Cell

## Abstract

We developed a simple, cost-effective smartphone microscopy platform for use in educational and public engagement programs. We demonstrated its effectiveness, and potential for citizen science through a national imaging initiative, *EnLightenment*. The cost effectiveness of the instrument allowed for the program to deliver over 500 microscopes to more than 100 secondary schools throughout Scotland, targeting 1000’s of 12-14 year olds. Through careful, quantified, selection of a high power, low-cost objective lens, our smartphone microscope has an imaging resolution of microns, with a working distance of 3 mm. It is therefore capable of imaging single cells and sub-cellular features, and retains usability for young children. The microscopes were designed in kit form and provided an interdisciplinary educational tool. By providing full lesson plans and support material, we developed a framework to explore optical design, microscope performance, engineering challenges on construction and real-world applications in life sciences, biological imaging, marine biology, art, and technology. A national online imaging competition framed *EnLightenment* *;* with over 500 high quality images submitted of diverse content, spanning multiple disciplines. With examples of cellular and sub-cellular features clearly identifiable in some submissions, we show how young public can use these instruments for research-level imaging applications, and the potential of the instrument for citizen science programs.

## Introduction

The microscope is perhaps one of the most symbolic instruments in science and microscopy, and one of the highest impact technologies in all of science. By providing the ability to directly observe the microscopic world, microscopy has underpinned discoveries across the breadth of all science disciplines. The history of the microscope shadows that of the modern scientific revolution ^1^. Arising in the 1600’s during the emergence from the dark ages, the microscope, its development and its revelations, helped lead the world into, through and then beyond, the scientific Age of Enlightenment. Seminal work by Robert Hooke ^2^ and others in the 1600’s showed, for the first time, the microscopic world and laid the pathway to our modern deep understanding of the cell and the fundamental building blocks of life and disease. The modern microscope has evolved to include, amongst others, phase contrast, darkfield ^3^, fluorescence ^4^ and super-resolution methodologies ^5^. It is a complex instrument combining advances in optics, detection, light generation, computation and engineering. However, what is remarkable is that despite these advances, the microscope remains at its core the same, simple, compound lens optical instrument developed by Hooke and his contemporaries nearly 400 years ago.

All optical microscope platforms, from a standard widefield, to the scanning confocal, STED and others are based on a high power, short focal length objective paired with a low power, long focal length eyepiece lens ^6^. It is a simple, elegant and extremely powerful concept. Unfortunately, despite all this significance and underlying simplicity, the principles and impact of the microscope remains distant for not only most members of the public, but in fact many of the research-based end users. Although public access to microscopy, either through school, or via commercial toys and educational products, has increased in recent years, it remains limited for many. Where access is obtained, the focus is on the application of the microscope, disengaging the public from the principles of function and in doing so from much of the impact.

To address this disengagement, we developed a national educational microscopy imaging challenge for 12–14 years olds based around a smartphone microscope platform. Our objective was to create a smartphone imaging platform that was accessible and engaging to the public, whilst simultaneously producing the highest quality images possible.

The smartphone microscope is an instrument that pairs external objective optics with a smartphones’ built-in camera lens, camera and display screen to produce a simple microscope platform. It is not a new concept and several examples of varying complexity have been developed for different applications ^7, 8^. This includes uses in microscopy ^9– 11^, for educational purposes ^12^, for safety inspection ^13^ and to develop cheap, portable clinical tools ^14, 15^. The potential of the smartphone microscope for many applications is clear, but the largest impact is in its ability to engage the public through the smartphone interface. Feedback from educators and science communicators has shown the difficulty of use of traditional microscopes, with failure to quickly see an image leading to disillusionment and lack of interest in a younger audience. A smartphone microscope addresses this difficulty, and allows the educator to see what the pupil is seeing.

By combining the correct design and choice of objective for the microscope platform, we created a simple, cost effective system that can act as a highly effective, multi-disciplinary educational tool for exploring all aspects of microscopy. Our imaging program, *EnLightenment*, distributed these microscopes to 1000’s of 12–14 year old pupils and engaged them with multiple educational microscopy challenges. *EnLightenment* set out with definitive learning objectives for the pupils that included 1) Being able to image cellular and sub-cellular sized features using a standard smartphone; 2) Develop an understanding on how a microscope works; 3) Explore the diversity of microscopy applications across science, engineering and art. Finally, we set out to evaluate the effectiveness of the program and quantified feedback demonstrates our success.

## The smartphone platform

The smartphone can be regarded as the pinnacle of consumer technology of the modern Information Age. A standard smartphone combines a portable computer with a digital camera, a high-resolution display, a range of remote sensors, audio-visual interfacing and of course remote internet access and interconnectivity. This is all contained in an accessible package with constantly evolving software and associated applications. It is easy to see why the smartphone has become established as a standard everyday item for the majority of individuals across the developed world. Nearly 1.5 billion smartphones were sold in 2016 ( http://www.gartner.com/newsroom/id/3609817), and smartphones now dominate communication, socialising, information retrieval, work and entertainment ( http://mobilebusinessinsights.com/2016/06/twenty-surprising-mobile-stats-for-2016-the-smartphone-takeover/). In the UK alone, it was estimated that 71% of the 2016 population owned a smartphone ( https://www.ofcom.org.uk/about-ofcom/latest/media/facts)

On the one hand, the technology, and instant access to global information and discussion, could make the smartphone a powerful platform available to engage the public with science. However, one disadvantage of the smartphone revolution is the increasing challenge to disengage from the technology, and its primary use as a social media and communication platform: smartphone addiction ^16^. This is increasingly prevalent amongst young people in the 12–16 age group, which is a prime target age for generating and maintaining the impact of public engagement of the sciences ^17^. However, this can be used to an advantage, and if the smartphone itself is integrated into the educational activity, public engagement can be increased, and interaction maintained for longer periods.

An often overlooked fact is that the smartphone evolution offers a standardised reflection of cutting edge developments in electronics, global communication, data access and computation. Technology that underpins the advances in modern scientific research are reflected and in many ways mirrored in an everyday device accessible to the public. This is enhanced with the regular handset upgrades that are common with users. The public are constantly in possession of state-of-the art technology, through which scientific advances can be easily discussed. Again, this can be a key advantage when integrating the smartphone with a scientific or technological based public engagement activity.

## Smartphone microscope design and characterisation

Our smartphone microscope was designed specifically as an accessible, public engagement platform. In addition to usability and practicality, key elements are maximising the optical performance and providing an educational tool to demonstrate how a microscope functions. Our design is therefore based on three core principles: 1) Maximum optical resolution and image quality; 2) Functionality and ease of use for children and; 3) Kit-based form to enable a hands on impression of how microscopes function. Our aim was to produce a low cost (<$10) instrument capable of imaging single cells (sub 10 μm resolution) to features in larger mm sized objects, such as insects or jewellery.

### Instrument design

**Figure 1.**
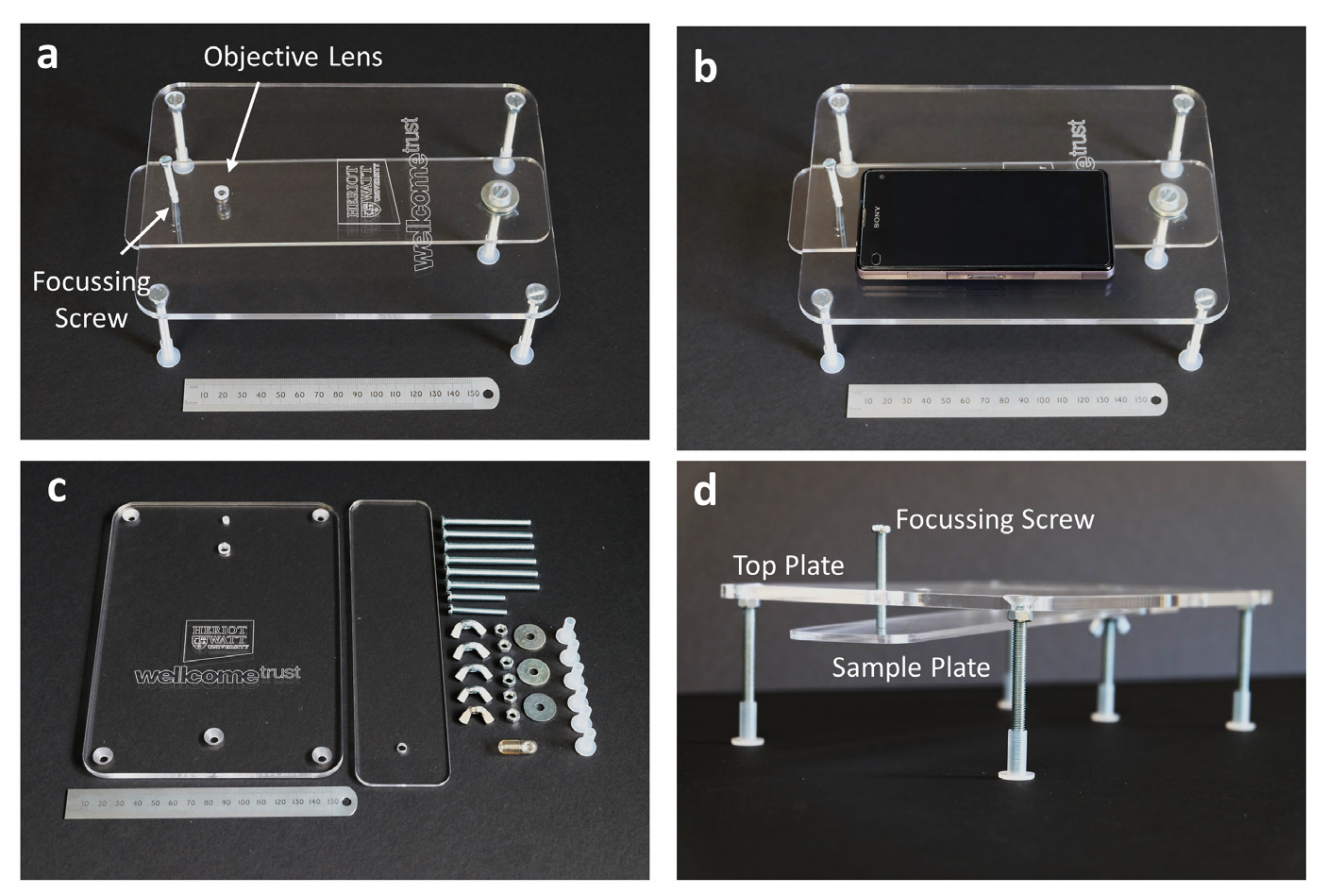
The smartphone microscope. ( **A**) The fully constructed smartphone microscope platform. With ruler for scale. ( **B**) Shown with a Sony Xperia Z1 compact smartphone for reference, with the smartphone camera overlaying the objective lens. ( **C**) A deconstructed microscope showing all the component pieces typically sent out in one kit. ( **D**) A closer look at the focussing mechanism and sample plate.

The design is based on a prototype unit built and developed as part of a SSERC supported STEM Education Support Officer placement. The final design is shown in Figure 1. The smartphone sits on a 210 mm × 150 mm × 5 mm transparent Perspex plate, which houses a single aspheric objective lens, positioned centrally to the short axis and off centre to the long axis, allowing for space for a typical smartphones camera to overlay it ( Figure 1b). Four countersunk 60 mm M5 bolts act as legs. A narrower 80 mm × 220 mm × 3 mm Perspex sample plate sits below this, held in place by an M5 bolt at the rear of the plate, which acts as a further leg support. Focussing is provided by a single 35 mm, M4 screw threaded through the base plate by the objective. Manually turning this screw puts pressure on the sample plate, angling it against its inherent tension to lower and raise the plate ( Figure 1d). Large washers provide tunability of the sample plate by increasing the distance from the objective, particularly useful for thicker samples. The microscope was supplied to pupils in kit component kit form ( Figure 1c) and includes a selection of nuts, wingnuts and washers to encourage an interpretive build.

The transparency of the Perspex serves dual purposes. First, it enables sample illumination, either from the smartphone’s built in LED flash/torch, or from a small LED lighting unit placed beneath the focussing plate. Second, it provides visual access to the microscope, which is important to connect the pupils to the operation of the unit. Prior to mass roll-out, the physical design was trialled in several local school and science festivals, with positive results. Together the component parts, when manufactured and supplied in bulk, amount to approximately $5 per unit.

### Lens selection and optical performance

It is a common misconception that magnification is the primary parameter for microscopy. In fact, maximising magnification can be detrimental, it reduces field of view and can lead to decreased signal and image quality ^18^. The key parameters for maximising image quality are in fact optical resolution and the ability to sample the image. Providing there is adequate signal to noise, it is these parameters that enable the user to distinguish small features. This is known as Nyquist sampling ^19^. For traditional imaging modes, and in the absence of optical aberration, resolution is dictated by the objective lens, as defined by the well know Rayleigh criteria ^19^:

$$\Delta x,\Delta y=\frac{\text{0.61}\lambda }{NA}$$

Where λ is the wavelength of the imaged light and NA the Numerical Aperture (power) of the objective lens. In high end microscopy, NA is typically 0.8–1.4, and resolution typically 350 – 200 nm for visible light, facilitated by high refractive index immersion media and complex multi-component compound objectives to minimise aberration and maximise throughput. Conversely, single lens aspheric lenses typically have NAs ranging from 0.1 to 0.8 and offer theoretical resolutions of 3 μm – 350 nm. In consumer and public microscopy, high end objective lenses are prohibitively expensive; however the principle of maximising NA to maximise resolution is conserved. Unfortunately, optical aberration, poor signal to noise, and image artefacts can significantly reduce practical resolution when compared to the ideal, and care must be taken to select optics that balance final performance with cost and practicalities.

In addition to optical aberrations, it is also important to consider the practicalities of high NA objectives. As NA increases, focal length and working distance, the absolute distances between the lens and sample, both decrease. The sensitivity of the focus also increases dramatically. This imposes practical difficulties in obtaining best focus, in positioning the sample and limits sample mounting to very flat architecture. The high precision sample stages and focussing mechanisms available in high-end microscopy are not viable for low cost consumer systems.

To balance performance with usability, we tested a variety of commercial single component aspheric objective lenses. A single aspheric lens offers a low cost, small footprint solution compared to compound objectives. These are what are typically found on smartphone microscope cameras. However, they can impose significant spherical and chromatic aberration away from the design wavelength, along with major off axis distortions. To balance performance with usability, we tested lenses with NAs of around 0.3–0.5, with a minimal working distance of several mm.

We selected 5 lenses for comparison; 4 commercial aspheric lenses from Thorlabs and a low cost, mass produced plastic aspheric of the type often used for ultracheap products (e.g. budget laser pointers) and found in many competitive smartphone microscopy solutions. Details of the lenses tested are shown in Table 1.

**Table 1.** Lens specifications. The cost is per bulk order (purchase of 100), individual cost for each lens is roughly twice this per unit. Specification of Thorlabs lenses taken from Thorlabs website. Specifications of budget lens where measured manually and subject to small error. NA, Numerical Aperture.

Lens	Supplier	Cost ($)	Material	NA	Focal length (mm)	Working distance (mm)	Diameter (mm)	Clear aperture (mm)
C170TME-A	Thorlabs	60	C0550 Glass	0.3	6.16	4.38	4.72	3.7
CAW110	Thorlabs	6	COC	0.19	10.92	9.33	6.28	3.4
CAY033	Thorlabs	6	Acrylic	0.4	3.3	2.0	7.4	2.7
CAY046	Thorlabs	6	Acrylic	0.4	4.6	3.0	7.4	3.7
Budget	Unknown	<1	Unknown	0.19	14	12.4	6.95	5.2

The C170 lens was used as a benchmark - a maximum quality, research grade glass aspheric designed for 780 nm, but with good performance over the visible spectrum. It was never intended for inclusion in the final product due to its high cost. The CAW110, CAY033 and CAY046 lenses were selected from a large selection of candidates based on prior experience and quoted specifications. The budget lens was acquired from a low cost commercial microscope toy of limited performance. Several budget lenses of similar specification were trialled, each delivering comparable results to the one presented here.

**Figure 2.**
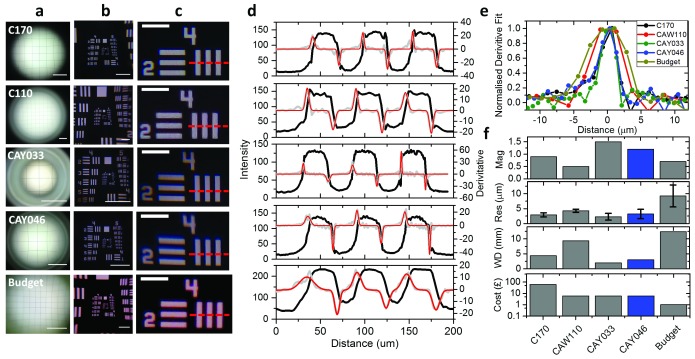
Optical performance and lens choice. The performance and imaging limits of 5 test lenses are quantified. Lenses C170, C110, CAY033 and CAY046 are all high quality aspheric lenses from Thorlabs. The Budget lens is a non-descript low cost lens from unknown supplier. ( **A**) shows cropped images of a 500 μm distortion test chart. Each image is 3215×2945 pixels, scale bars 1000 μm. ( **B**) and ( **C**) show cropped images of a standard USAF test chart. Column (b) is 1500×1500 pixels, scale bar 500 μm, column (c) is 500×320 μm, scale bars 150 μm. ( **D**) shows line profiles (black) through the selections shows in red in (c), along with first order differentials of those profiles (grey) and the associated multi-peak gaussian fits (red). ( **E**) shows a single example differential peak from (d) for each lens, normalised and overlaid at zero distance. This shows the measured diffractive limited focus for each lens. ( **F**) shows the measured lens magnifications, calculated from (b) and (c), the average FWHM of the differentials (Res) in (d) and the manufacturers quoted working distance (WD) and cost per unit for each lens. WD is measured for the budget lens. All data taken with a Sony Xperia Z1 compact using maximum camera resolution.

Characterisation of the lenses is presented in Figure 2. Testing was done using a Sony Xperia Compact Z1 smartphone and Open Camera app (Mark Harman, V1.39) to access the full camera resolution. Comparable results were achieved with other leading camera models at the time, including the Apple iPhone6 and the Samsung Galaxy S5.

Figure 2a shows a column of images of a 500 μm period grid distortion target (Thorlabs, R1L3S3P) taken with each lens. These same images are shown larger, and alongside similar images for a 100 μm distortion chart in Supplementary Figure S1. In all cases, a white light LED was used to illuminate the samples from below, overlaid with a single sheet of tissue paper to act as a diffuser. Clear off axis aberrations are present in all images, predominately pincushion, which is expected for short focal length high power aspherics when imaging in widefield. Edge effects are also apparent in the 4 plastic lenses, due to a decreased clear aperture of each lens over the smartphones camera lens. The known parameters of the distortion chart data allow for accurate determination of magnification in both spatial directions, using details of the IMX220 Sony Image Sensor in the Xperia Compact Z1 (1.2 × 1.2 μm pixel size). Magnifications for each lens are shown in Figure 2f (top panel).

Magnification varies from 0.5 for CAW110 lens to 0.7 for the budget lens, 0.9 for C170, 1.2 for CAY046 and 1.5 for CAY033. Smartphone microscopes are not high magnification systems. A smartphone aspheric lens, the eyepiece lens, will have a focal length of a few mms due to design restrictions of the slim smartphone cases. (The focal lengths quoted on smartphone cameras, typically between ranges of 18–60 mm, are equivalent focal lengths to produce equivalent images on a standard 35 mm format sensor). Consequently, when paired with a similar focal length objective magnification, which is given by the ratio of the two lenses, will be around unity. However, as discussed previously and shown below, this is not a limiting factor, due to the small pixel sizes of smartphone cameras allowing for adequate image sampling.

Figure 2b shows images of a standard USAF test chart, imaged using a white light LED as described previously. Figure 2c shows a zoomed in region of the images on Digure 2b. To assess optical resolution, often the smallest discernible feature on a calibrated test chart, such as the USAF chart, is used (see Supplementary Figure S2 for examples). However, by taking cross sections through a USAF element a precise measurement of the width of the optical point spread function, and thus absolute resolution limit, can be determined. Figure 2d shows the cross-sections through element 4-2 (element width 27.84 μm) as shown in red in Figure 2c. As the USAF test chart is a binary chrome-on-glass pattern and the chrome to glass edge transitions can be considered ideal step changes, the first derivative of the cross section returns directly the optical point spread function profile ^20^. These derivatives are shown as grey on Figure 2d, along with the Gaussian fit for each transition as a first approximation to the point spread function (PSF) Airy function. Figure 2e centres and overlays one example, including the sampled pixel points, of each transition derivative for each lens. The average and standard deviation of the 6 Gaussian widths are shown in Figure 2f, second panel, for each lens, long with the working distance and cost per unit for each lens type. Supplementary Figure S2 reproduces these overlays for each lens, relative to CAY046 lens for comparative reference.

The PSF width in these idealized tests varies from 2.3 μm for CAY033, to 2.9 μm for C170, 3.2 μm for CAY046, 4.3 μm for CAW110 and 9.2 μm for the budget lens. Taking the accepted Nyquist sampling limit that roughly 7 points are required to accurately fit and describe the central peak of an in focus PSF, the cross sections in Figure 2e show that the low magnification, but high resolution, of the CAW110 lens slightly undersamples the PSF (5 points in PSF), whereas the other lenses are all oversampled. This demonstrates that despite the low magnifications, for these four lenses, the smartphone microscope can fully and adequately sample all information down to the diffraction limit. However, in practical terms, it is unlikely that all but the most robust imaging challenge would be hindered by the undersampling of the CAW110 lens.

Smartphone microscope design must compromise between cost, functionality, and performance. For example, CAY033 gives highest resolution, but has severely limited field of view and a very restrictive working distance that limits usability. Balancing the field of view ( Figure 2a) with resolution and sampling ( Figure 2e), and cost and working distance ( Figure 2f) led to the selection of the CAY046 as our lens of choice (highlighted in blue in Figure 2f). It delivers approximately 3 μm optical resolution with a manageable working distance and large field of view. We trialled several of these lenses at public science festivals prior to the final selection, in prototype smartphone microscope platforms, with various smartphone models. The public confirmed our choice in terms of ease of use and quality of imaging, and we used these trials to verify that a lens with a degree of off-axis aberration and edge effects was in fact a positive. These effects both frame any images, and allow for a degree of artistic flexibility, which the public found interesting and engaging.

It should be noted that exact performance will depend on the smartphone of choice, as camera, camera lens and general performance vary across models. As the smartphone microscope is not an ideal, infinity corrected microscope system, the phone’s autofocussing and camera body, which separates the camera lens from the objective lens, have an impact on the values presented. However, multiple trials confirmed a largely consistent result to those presented here, under normal conditions where the smartphone lies flat and directly on the microscope body. No special considerations were taken over the selection of the Sony Xperia Compact Z1 as a trial camera of choice.

**Figure 3.**
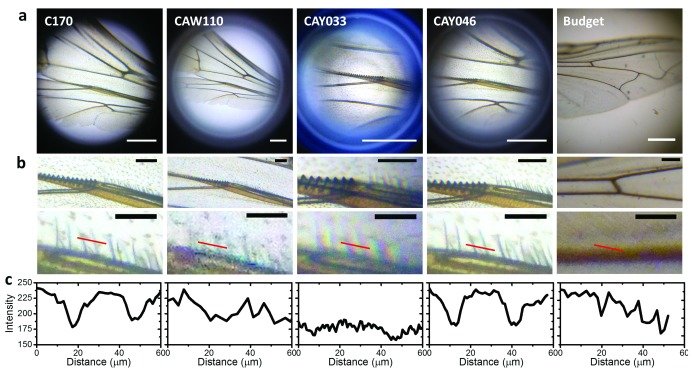
Example imaging. Imaging tests using an insect wing. The same wasp wing and same field is used for lenses C170, CAW110, CAY033 and CAY046. A separate wasp wing was used for the budget lens. ( **A**) shows cropped images from each lens. Each image is 3400×2952 pixels, scale bars 1000 μm. ( **B**) shows zoomed in regions from row (a). **Top row** each image is 824×336 pixels, scale bar 200 μm, **bottom row** each image is 300×150 μm, scale bar 100 μm. Individual hairs clearly visible for some lenses. ( **C**) shows line profiles through the selections shows in red in (b). Individual hairs are easily identified in C170 and CAY046, but not with any other lens. All data taken with a Sony Xperia Z1 compact using maximum camera resolution. CAY033 and Budget lens images have been white balanced adjusted using an ImageJ macro by Vytas Bindokas; Oct 2006, Univ. of Chicago // Modified by Patrice Mascalchi, 2014, Univ. of Cambridge UK.

To place the above results in an imaging content, Figure 3 shows the same wasp wing sample imaged using the first 4 lenses, and a separate wing for the Budget lens (due to sample degradation the same wing was not available throughout). Figure 3a shows the entire field of view as seen by each lens. Figure 3b shows two zooms of the wing, the top row an area of 824 × 336 pixels, the bottom row and area of 300 × 150 μm. Figure 3c shows cross sections through the red marked lines of Figure 3b.

The C170 lens delivers optimal performance, with the largest, flattest field of view and distinct hairs, shown here to have width 6–7 μm, clearly identified and resolved with good contrast in Figures 3b and c. The CAW110 lens delivers poor absolute imaging, reflecting the reduced optical resolution (4 μm). Despite similar performance characteristics between CAY033 and CAY046, shown in Figure 2, CAY033 performs badly in this test. There is reduced optical throughput, and artefacts in the image, which reduces signal to noise and degrades the final images in Figure 3b. A primary reason is the high sensitivity to focus of this lens, making it challenging to achieve optimal results with the focussing method employed. Despite a different sample, the budget lens can clearly be seen to perform badly. Image quality is suppressed across the field, with only larger features identifiable. Figure 3 thus confirms the selection of the CAY046 lens as the optimal balance between cost and image quality.

## EnLightenment

**Figure 4.**
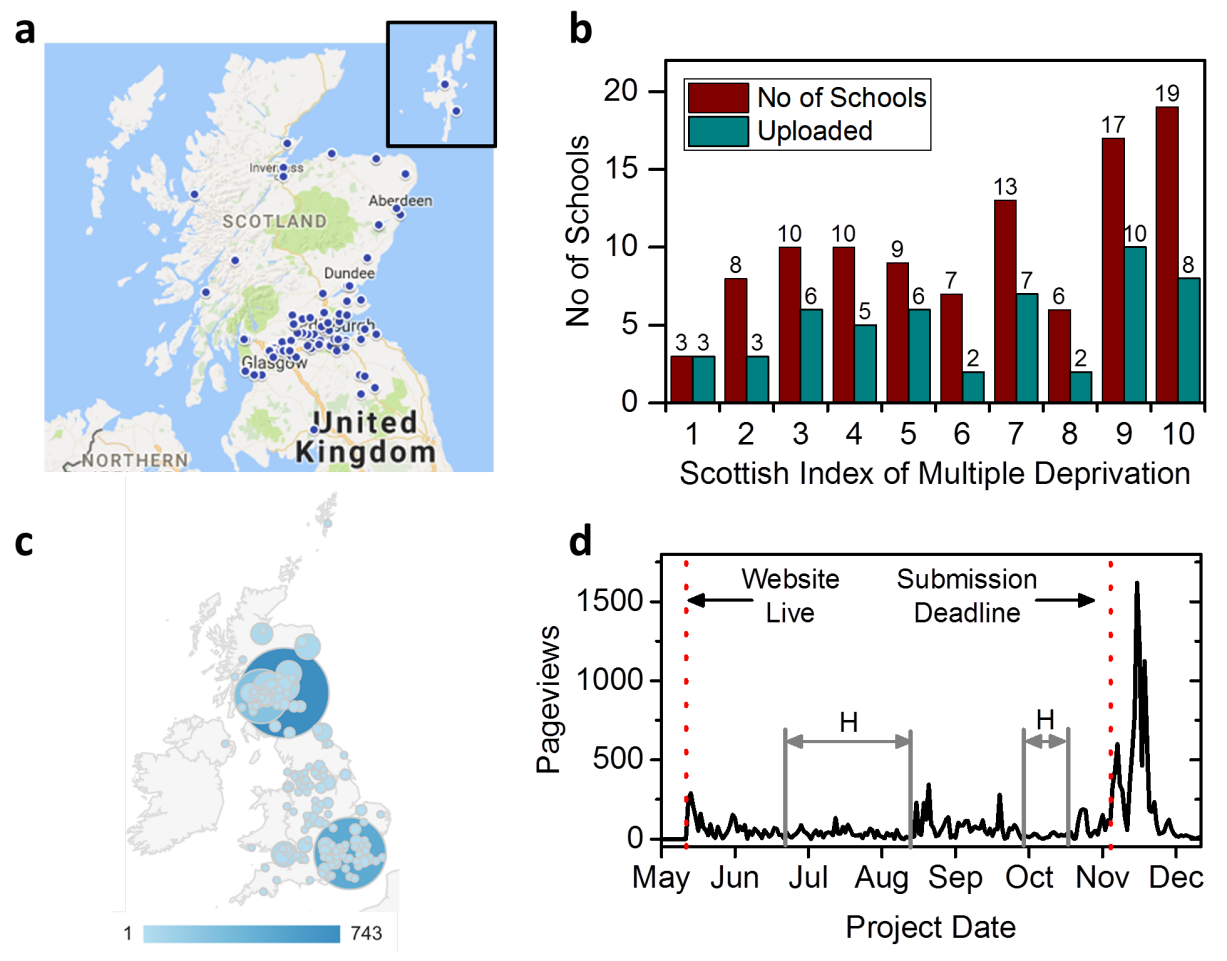
*EnLightenment* geographical spread, image upload and website activity. ( **A**) The location of all schools that received *EnLightenment* smartphone microscope kits. ( **B**) Number of schools that received kits, and the number that uploaded images to the *EnLightenment* website, according to the Scottish Index of Multiple Deprivation Index (SIMD). SIMD identifies areas of poverty and inequality across Scotland, with a lower score represents most deprived. ( **C**) The UK geographical spread of website activity over the duration of the program. ( **D**) Distinct pageviews for the *EnLightenment* website for the duration of the project, highlight time period of Scottish school holidays (H).

The *EnLightenment* project sent 510 of the smartphone microscopes, each with a single CAY046 lens, to 102 secondary schools throughout Scotland. Almost all kits were sent out early in the school year (September 2015). Each school received 5 microscopy kits with associated lesson plans and guidance (see Supplementary File 1). Prior to school selection, all secondary schools in Scotland were contacted with information on the project and submitted their interest via an online form. Direct contact with teachers was established via e-mail, with teachers at events such as science festivals and via established on-line networks such as the Institute of Physics. As demand for the kits outweighed resource, schools were selected for the program based on school motivation, balanced with a national geographical spread, and covering a range of poverty and inequality regions, as identified by the Scottish Index of Multiple Deprivation ( SIMD). Figure 4a shows the location of each of the schools involved in the project. Figure 4b shows the number of schools who received kits from each SIMD grouping.

The project based its roots in biological imaging but did not restrict schools to these fields; interdisciplinary activity was encouraged. In addition to combining physics and engineering in the system design, schools were free to use the microscopes for whatever application they desired. To encourage engagement, the project centred around a national imaging competition. Students were invited to upload their images to our bespoke website, where upon closure of the competition, after 3 months, winners would be selected by an expert panel and prizes (valued between £100–1000 for both pupil and school) awarded at a formal ceremony. All uploaded images were visible by any website visitor in a metadata-tagged gallery. Figure 4b shows the number of schools that uploaded images during the competition for each SIMD index grouping. Over 500 images were uploaded to the website, from 52 schools.

The *EnLightenment* website also offered lesson plans, suggested sample preparations, teacher resources, student resources, and background information on microscopy and its use in modern life science research. Some examples are given in Supplementary File 1. All lesson plans and information were prepared in conjunction with teachers, and in alignment with the Scottish Government Curriculum for Excellence. The website attracted over 20,000 unique page views in 12 months, primarily from across the UK ( Figure 4c and d).

A series of public lectures and demonstrations supported the programme, beginning with an opening event at Our Dynamic Earth in Edinburgh (a fixed public science space) and culminating in the largest of the Edinburgh International Conference Centre ‘Innovation Nation’ series of talks. These brought the *EnLightenment* message to the widest possible constituency. The *EnLightenment* team also visited over 20 schools directly during the project. The awards ceremony formed part of the Scottish closing ceremony of the International Year of Light (IllumiNations). As part of the project, over 12,000 additional members of the public, teachers, and academics were reached in the 12-month period.

### Submitted images

The uploaded images demonstrate two core results. First, the diversity, inspiration and capabilities of 12–14 year olds who are engaged with science. Secondly, the potential and wide-ranging applications of a smartphone microscope engineered for simplicity and optical performance. Supplementary Figures S3a–d show collages of all uploaded images, and full size versions are accessible via the *EnLightenment* website. Subjects vary from insects and wildlife to plants, electronics, art, chemistry and engineering. Images were uploaded from a variety of devices covering most major tablet and smartphone manufacturers and models of the time period. Many submitted images clearly resolved sub-cellular structures. A prime example, resolving plant cell walls and nuclei, can be seen here; Iodine-on-onion submission. These examples show how sub-cellular features can be explored with the smartphone microscope in a standard classroom with minimal sample preparation.

**Figure 5.**
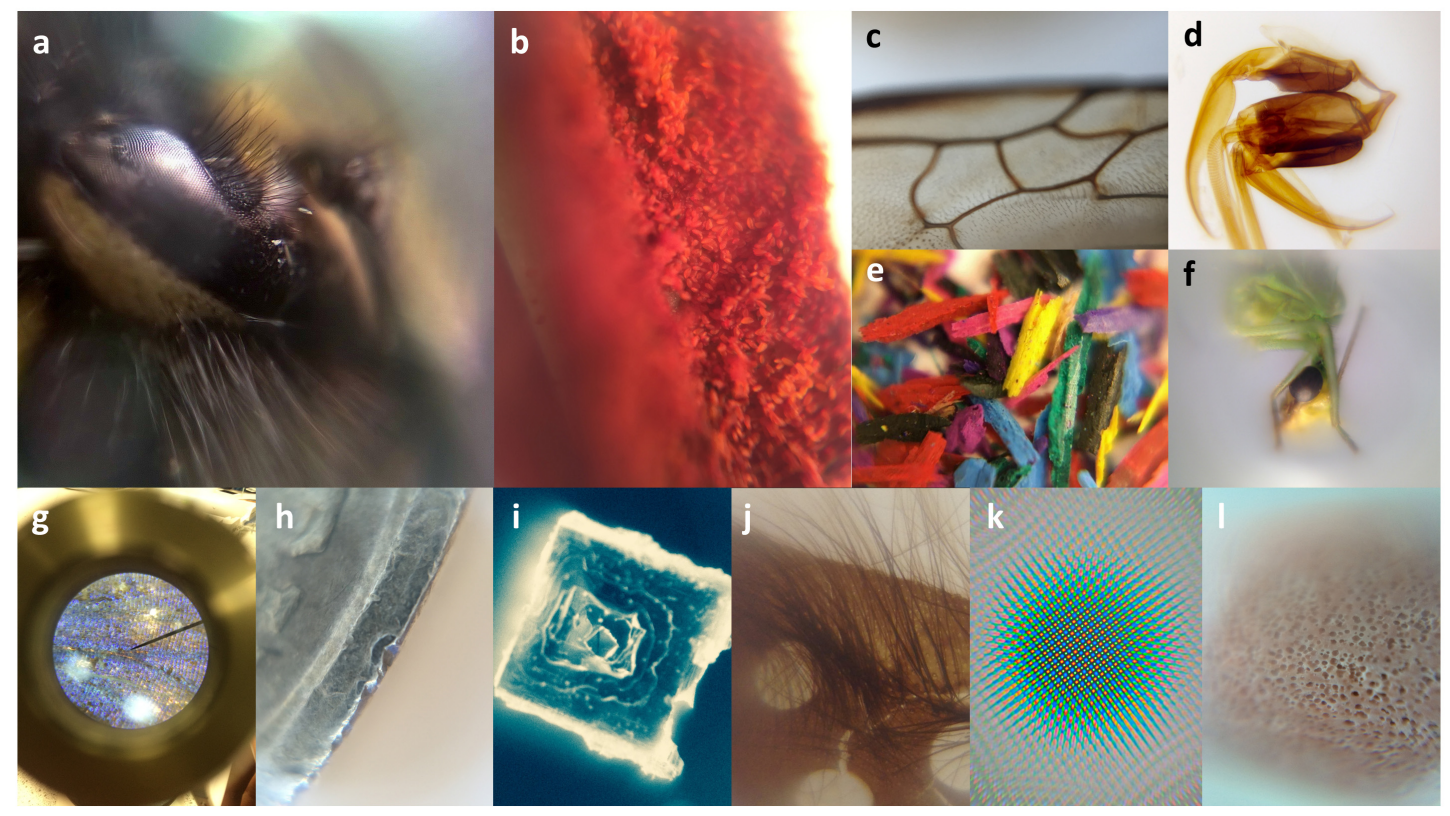
Winning *EnLightenment* images. The winning images from the *EnLightenment* competition, selected independently of school, from over 500 submissions by a panel of scientists, artists and public engagement experts. ( **A**) The overall winning image – “Head of a Wasp” from Fortrose Academy ( **B**) The runner-up image – “Pollen Grains on Anther” from Alva Academy. ( **C**)–( **L**) show the remaining finalist images. ( **C**) “Wasps Wing” from Dollar Academy. ( **D**) “Bee Mouth” from Alva Academy ( **E**) “Entwined Pencil Sharpenings” from Galashiels Academy. ( **F**) “Eye of a Lacewing” from Waid Academy. ( **G**) “Buttterflies Delight” from Dunblane High School. ( **H**) “On the Edge (of a £ coin)” from St Andrews RC Secondary School. ( **I**) “Sodium Chloride Crystal” from Robert Gordons College ( **J**) “Bee Skin” from Galashiels Academy. ( **K**) “Astounding AMOLED” from Currie Community High School. ( **L**) “Porous Pencil Tip” from Queen Anne High School.

The winning images were selected by a panel consisting of an academic public engagement specialist with a background in marine biology (LCW), a cell biologist (RRD), a biophysicist (PAD) and a microscopist (Stephen Webb, STFC, Rutherford Appleton Labs, UK). We increased diversity in the judging panel with the inclusion of a representative from the visual arts world (Hannah Imlach, Artist in Residence Heriot-Watt University) and a Director of a public science exhibition space (Hermione Cockburn, Our Dynamic Earth, Edinburgh, UK). Image quality, artistic merit, and subject content were all considered. Figure 5 shows the winning images, with Figure 5a the overall winner, Figure 5b the runner up and Figure 5c–l the other finalists.

The winning image ( Figure 5a) shows a wasp eye taken with a Nokia Lumia 635 phone (image from Fortose Academy). Individual facets (typically 10–20 μm in diameter) are clearly visible as are individual hairs and other features. Great care has been taken to include depth in the image, along with individual small features. The off-axis aberration of lens CAY046 acts as a frame to focus the eye and the content to the centre of the image, something that was common in many uploaded images and that proved a key feature of interest for many pupils and the public. The runner up, Figure 5b, shows individual pollen grains on anther, taken with an iPhone 6 (Alva Academy). No details of the pollen type were provided, but individual grains are likely a few 10’s of μm in size. The other finalists were selected based on image quality and artistic merit. Supplementary Figure S4 shows zoomed in images of a select few of the winning images for further detail, demonstrating the optical quality of these finalist images. Note that as the microscope offers a degree of flexibility in its use, details of the exact configuration used for each image are not known and magnifications cannot be accurately included on these images.

There is a consistently high standard across all submitted images. Importantly, the feature sizes resolved here are comparable to those acquired in our controlled physics laboratory environment (i.e. Figure 2) – this confirms that the general public can assemble and use our smartphone microscopes optimally. Together the finalists, and other submitted images, demonstrate that the smartphone microscope presented here is capable of imaging individual cells and other micron sized objects with multi-colour clarity and a depth comparable to modest commercial microscope systems.

### Feedback

For each school that received the kit, feedback was requested via a web form from participating teachers. Questions focused on interdisciplinarity, aspirations and experience of pupils, as well as requests for improvements to the kits. We received detailed feedback from 17 schools with the response overwhelmingly positive. Most returned scores of 4/5 or 5/5 for questions on usefulness of the kit, communication in the program, how activity inspired pupils and appropriateness for target age group. Feedback also asked about usefulness of the microscopes for cross-disciplinary work. There was clear bias towards biological sciences, but notable applications towards physics, art and others. Results are shown in Supplementary Figure S5. Supplementary File 2 shows a full collation of this feedback, including all comments received from the schools exactly as submitted via these forms. Although overwhelmingly positive, many valid points where raised for minor improvements. Some are non-viable due to cost or practicality (better lenses, collapsible legs, more complex focusing etc), but we have already made modifications to the position of the objective lens for follow on projects and other small changes.

In addition, audience-appropriate evaluation was conducted throughout the *EnLightenment* project, devised for each associated supporting activity. For each event, audience feedback and metrics were collated, for example during the Dynamic Earth event in 2016 ( Supplementary Figure S5b), via feedback forms, social media and attributable quotes. The response was overwhelmingly positive throughout. Of particular note is that before the activity no member of the public could describe how a microscope actually works, after the activity 77% of people could comfortably explain microscopy as a system of two lenses.

## Conclusions

We have shown how a simple and low-cost smartphone microscope system can deliver high end optical imaging. We have carefully selected the design and optical components to maximise performance, whilst retaining a cost effective and engaging platform to maximise usability for secondary school pupils. Our kit based system provides engineering challenges, and allows pupils to study directly the component parts of a functional microscope. Our *EnLightenment* program demonstrated the effectiveness of this approach, and we received research grade image submissions from school students of remarkable variety and quality.

Based on the success of *EnLightenment*, it is clear this is a platform for wider ranging citizen science and further educational programs. With little modifications, we have translated the platform in this work to a new NERC funded national imaging initiative coined “She sees sea beasties on the seashore” that take these microscopes to the Scottish coastal waters for a primary school targeted citizen science program to identify and catalogue plankton ( http://enlightenment.hw.ac.uk/seabeasts/). This identifies the potential of the instrument, and further dissemination of this project will follow in due course.

*EnLightenment* was a considerable success in terms of pupil and public engagement, public interest and quality of submissions. This is largely due to the optical capabilities of the instrument and a complete and structured support program that ranged from design to characterisation to dissemination. However, the inclusion of the smartphone as the key component, not only to take the pictures but for upload, geotagging, metadata input and engagement with the website, played a significant role. The public, and school pupils are clearly capable of engaging with and delivering high end science, but it must be complimentary to modern lifestyle and associated technology.

## Project approval

No ethical concerns were identified, meaning approval was not required to engage the schools in the project. Participant data were removed from image entries before publication. The project was designed in collaboration with a school teacher (Bryce) who was seconded to the Scottish Schools Education Resource Centre, to ensure our programme was aligned with the Scottish Government Curriculum for Excellence.

## Data availability

Raw data for this study are available from OSF, http://doi.org/10.17605/OSF.IO/D9E2J ^21^. Each raw data set title corresponds directly to the publication figure label. All image data was analysed using ImageJ (V1.51j8) and only adjusted for brightness and contrast to aid publication clarity, unless otherwise stated in text. All numerical data was processed in OriginPro 2016 SR2 for graphing, statistics and fitting.

## Supplementary material

**Supplementary File 1: Example lesson plan.** Created to aid teachers in introducing the smartphone microscopes into the classroom. Plans were aligned with the Scottish Curriculum for Excellence’s Experiences and Outcomes (E’s and O’s) for Sciences. Further PowerPoint presentations and supporting documentation are available on the website.

**Supplementary File 2: Responses collected and summarized from teacher feedback surveys.**

**Figure S1: Distortion test targets.** A grid distortion target (Thorlabs RL3S3P) allows for magnification, field of view and barrel, pincushion and astigmatic aberrations to be assessed. **Top Row** images with each of the tested lenses of a 100 μm grid distortion test chart. **Bottom Row** images with each of the tested lenses of a 500 μm grid distortion test chart. Each image is uncropped horizontally, but cropped vertically, to show maximum field of view on the test smartphone (Sony Xperia Z1 compact). The high end C170 lens shows minimal edge effects and reduced off-axis distortion. CAW110, CAY033 and CAY046 all show varying degrees of edge effects but similar aberrations. The Budget lens shows significant edge effects and aberration throughout the image.

**Figure S2: Further resolution tests.** Visual and quantified assessment of optical resolution with each test lens. **Top Row** zoomed in view of elements 7-6 (width 2.16 μm) and 6-1 (width 7.81 μm) taken with each lens. Often the ability to visually distinguish between elements of a test chart is used to determine resolution. However, this is highly dependent on signal to noise and subjective to interpretation. However, it is clear that the choose CAY046 lens outperforms the budget lens, and CAW110. **Bottom Row** the same measured point spread function results shown in Figure 2e but separated to show the differential profile for each lens, alongside the Gaussian fit and the CAY046 profile for reference.

**Figure S3: Submitted images.** All submitted images to the *EnLightenment* website (510 in total), shown in no particular order. Full details can be found at http://enlightenment.hw.ac.uk/.

**Figure S4: Zoom in on winning *EnLightenment* images.** Zoom in on selected winning images from the Enlightenment competition shown in Figure 5. (A) “Head of a Wasp” (B) “Pollen Grains on Anther” (C) “Astounding AMOLED” (D) “Buttterflies Delight” (E) “Entwined Pencil Sharpening’s”. (F) “Porous Pencil Tip”. Scale bars cannot be accurately determined as information on the camera and exact configuration was not always provided (and was not requested). However, the pollen grains in (b) are at most 10’s of microns across, and as an example individual red or blue AMOLED pixels in (c) are likely separated by 50–100 microns depending on the screen that was imaged.

**Figure S5: Feedback. Top Row**: feedback from survey sent to teachers after the *EnLightenment* program closed showing responses to questions on usefulness and engagement of kits (left) and how kits can contribute to, or be used, in various interdisciplinary subjects (right). **Bottom Row**: Feedback from audience members of a public science festival event held at Dynamic Earth, Edinburgh, April 2016. The public were asked (a) how much they learnt, and (b) their enjoyment in using the smartphone microscope platform.
