# Supplementary material for: EnLightenment: High resolution smartphone microscopy as an educational and public engagement platform
Source: Wellcome Open Res. 2018 May 3;2:107. Originally published 2017 Nov 6. [Version 2] doi: 10.12688/wellcomeopenres.12841.2 (PMC5861559; doi:10.12688/wellcomeopenres.12841.2)
Supplement: Supplementary file 2 [file wellcomeopenres-2-14682-s0001.tgz › 17f6740c-6ca9-4b6d-993e-66aade9aff07.pdf]

**Supplementary File 2**

**EnLightenment – Collated and Summarized Teacher Feedback**

17 Responses from 101 Sent Surveys

**Recommend**
87% would recommend to colleague, 7% would if changes made, 6% wouldn't

**Problems**
- Nuts and bolts came apart quite easily. It's a little fiddly putting the sample between the two perspex plates.
- Very difficult to manoeuvre samples in and out from under the lens (we ended up taking away one of the legs to make it easy to pivot the sample stage in and out).
- Not as easy to use with phones other than i-phones or with certain other devices (e.g i-pads, tablets)
- Should not have used superglue for the lenses. glue attacked the plastic
- We couldn't make them work in the way that I'd seen at the display. They magnified the image but certainly not to microscope levels. The instructions were unhelpful - it seems the designers wanted to leave it to the users to 'discover' how best to use the device. But I'm implementing the new Ad Higher and Higher course, whilst revising the N4 and N5 course and redeveloping the S1 and S2 courses. At this point I don't have time to mess about with kit - it either works or it doesn't. Sadly, this didn't work and neither me nor any of my colleagues had the time to invest in working on it.
- Difficulty focussing the images - depth of field caused issues
- Deciding which way the lens went
- Had to find some way of making sure the lens didn't fall out if pupils held the kit the wrong way.
- The main problem was identifying the correct orientation for the lens and then fixing the lens so it would not fall out when the kits were being used in class.
- I placed the lenses in the kits first before giving them to the S1 pupils to ensure they didn't get lost.
- Difficult to use
- Sharing amongst 20 pupils was not appropriate.
- Nokia 1020 phones have a camera lens placement that doesn't allow these phones to be used.
- Some smartphones (including my Nokia) had the lens in the wrong place to fit the microscope. We had to remove the focussing screw and move the plate manually to focus.
- There weren't any issues with the kits
- The hole for the lens is slight too close to the focus screw ... this stops the microscopes being used with some makes of android phones/tablets. Moving lens down 1 cm would remedy this issue.
- None at all. The kit was delivered in a very organised box.

**Improvements**
- A slightly more sturdy construction. I'm not sure if there would be another way to mount the sample and retain the simplicity of the design?
- The hole for microscope lens needs to be further away from the short edge of the stage (i.e more central)
- The sample stage could then be shorter so that it can be rotated clear of the device stage to make placing the samples much easier (this can be frustrating

- There need to be more holes machined into the top stage (one centrally but two at each side) which will allow an i-pad / other tablet to be positioned. This would be more useful for demonstrating to a larger group / whole class.
- Some way of introducing friction / grip on the surface of the perspex to help hold the phone and sample slides in place once set by the user.
- A higher quality lens (though it has to be said that selecting / preparing samples that would give good images was a good part of the challenge with the kit)."
- Include appropriate glue.
- Improve the instructions. The version I received seemed to refer to an earlier version of the kit. And include enough detail to make it fairly easy to assemble and operate.
- we used a magnet under the screw to help keep sample in place
- Some phones with central cameras didn't work
- Having some way of holding the lens in place.
- Having collapsible legs would benefit the storage options for the kit.
- see above - also move the lens hole away from the other screws for the focus as the camera position in some phones meant that they couldn't line up the camera with the lens because of the screws.
- More available to school.
- Can't think of any. Simple is best.
- Personally I would prefer them to be slightly smaller. the pupils only used them with phones and the kits could be made half the size.
- Also it would be useful to be able to buy replacement lens. One of our has rolled away and I am not sure what kind I need to replace it.
- If we could maybe get more microscope kits please...

**Quotes**

- *The kids loved it*
- *No improvements needed at all, simple is best*
- *Pupils really appreciated their use and enjoyed being able to record their image on their own phone / tablet.*
- *The pupils really enjoyed the activity. Also, the experience of entering the competition and attending the closing ceremony for the International Year of Light was a great experience for them. Other, more senior pupils also enjoyed using the microscopes and we are starting to consider other ways that we can utilise them throughout the school.*
- *All pupils loved the activity and shared the pictures around the school.*
- *They enjoyed the fact they could use their phones to take picture and could take it home to let others see what they had been doing*
- *Cool*
- *The pupils thought the microscope kits were really good as they could use their own phones in class as well as take a photo of the microscopic slide home.*
- *Really fun - it was great to see things in a different way. never knew there were holes in the dots on a pound coin.*
- *Pupils really enjoyed the activity and were keen to show their friends and parents the photo's they had taken.*
- *One pupil had slides made by his grandfather in the 1950s. He brought these in and the pupils really loved looking at them. The pupil was delighted to have photos of his grandfather's slides to keep.*
- *Pupils really enjoyed using them, lots of "wow look at this" comments. Liked the idea of putting them together themselves. The Haribos were a nice touch.*

- *They are great to use, engaging and intend looking for more opportunities to use them. The only issue is overcoming the school policy on mobile phones in class.*
- *Our pupils loved this activity and were proud of their results. They really enjoyed the challenge of building the kits up and enjoyed exploring everyday items using the microscopes.*
- *They thoroughly enjoyed it. Maybe a little video showing how to assembly it as the instructions were a little wordy for some pupils.*

**Future use**
Yes for same activity – 35%, Yes for different activity – 53%, Maybe – 12%

**Uses**
- Looking at crystals in chemistry, a multitude of different tasks in biology. Perhaps developing some analysis / CSI tasks.
- Run a similar competition within the college (involve science & art departments)
- Run a competition where pupils try to identify everyday objects from pictures taken using the smartphone microscope.
- STEM Club members undertake extended project to re-design smartphone stage.
- Generating images and short videos of ion migration experiments, crystallisation / precipitation etc to share digitally with pupils as part of delivery of curriculum.
- S6 pupils have used them to generate images of their crystalline products (e.g benzocaine crystals) from organic synthesis. They are ideal for this purpose as the pupils can easily generate high quality images for use in their investigation reports.
- As low power examining microscopes
- They could be used with our S1/2 in our Cells topic to look at different cells up close as well as further up the school with S4/5 in investigations in forensics.
- I passed the kits onto the head of science who told me that art and graphics had both expressed an interest in using the kits.
- We may try to integrate them in to courses at a later date.
- Might work with the HE department so that the S3 students can see the effect of temperature on the gelatinization of starch. Haven't tried this yet to see if the magnification is sufficient, but would allow students to video the process.
- Can easily substitute for standard microscopes. We used them with S1 looking at crystals produced from evaporation, pupils able to all see the sample, and easy to print out result which is not possible with other microscopes. Also used to look at protozoa with S2. Biologists looking to use them at various points in their course.
- looking at crystals
- We would like to use them to introduce sizes and scales to S1 and also for teaching cells. Currently it is very difficult to see if S1 pupils are looking at the right thing under a conventional microscope. these microscopes allow pupils to work collaboratively and point out features to each other.
- S1/S2 Cells course

**Alignment to CfE**
71% - Yes, 29% - somewhat

**Flexible in their use, easy to integrate into ICT.**
- We have mostly used them for generating images for the competition so far. The fact that the kit was slightly tricky to use and the pupils had to really explore how to get the best possible images was definitely in line with the principles of CfE. The pupils had to think outside the box, develop resilience and confidence in using the equipment, handling the samples etc to be able to produce the images they did.

- The pupils really enjoyed the whole feel of the task. I think the fact that pupils were involved in building the microscopes really helped them see the IDL aspect.
- Can be used for any part of the curriculum which needs a close look at structure
- The kit definitely made the pupils more effective contributors by working together to build the microscope as well as making them successful learners by being more engaged in the topic of cells.
- From an active learning perspective the project was a real hands on experience driven by the young people
- Excellent for practical demonstration of how microscope works
- Allowed for exploration of different topics in a new way.
- very relevant
- Useful to have as a resource that can be grabbed on the spur of the moment. Will use them later in the year for plant / cell studies.
- Can be used to great effect in all sciences
- Brilliant Cross curricular project, our enlightenment picture were added to the Art Department's Gallery that went on display in our Town Centre.
- They were used not only for this activity but also when teaching cells topic.

**Can we use as a transition project?**
- Yes, but surely depends on whether the kids have smartphones or not? Otherwise it would have to rely on schools having the digital equipment in place.
- The kits would be very useful as a transition project to inspire interest and allow pupils to understand that when they come to secondary it is not all about distinct subject. The only problem I would encounter using this as a transition project is the number of kits would not be enough for how the transition projects are set up.
- I think they would be a good way of having older pupils work alongside transition pupils.
- Very appropriate, however, most schools don't teach about cells till S1
- We have an S1 open afternoon. Current P7's and current S1 families are invited to the school to meet staff informally. We might use the kit at this event.
- Primary pupils would love them, we will be looking to use them in a number of areas, could easily take them to primary schools. Very easy to understand basics of how they work.
- I think this would be ideal!

**Satisfaction**
94% - yes, 6% - no

**Other quotes**
- I think it would be good to see some images taken by the university if you had any of things we would not be allowed in schools.
- We plan to use the microscopes across the sciences from S1-->S6.
- Just wanted to say thank you so much to all at Heriot Watt for a fantastic time at the Award / Closing Ceremony. We particularly enjoyed the interactive activities session in the morning. It was a fantastic introduction to some brilliant research projects and was perfectly pitched for the audience. The research and development team also made us feel very welcome and were most helpful throughout the day. The only disappointment for the pupils was that we didn't have time to do the lab tour because of our long journey, but we would hope to bring them back for another visit sometime!!
- Thank you very much for providing the school with this useful resource. The pupils really enjoyed using them and learnt a lot from the kit.

- Thank you for the kits, they are great and we will definitely use them each year with our S1 pupils.
- A fantastic resource, many thanks. Do you have any issues with us trying to copy them for our own use, not commercial? If no objections, can you give us a source or spec for the lenses as that is the one part we wouldn't be able to obtain ourselves?
- Thank you so much for our kits. We really appreciate them and use them regularly across the sciences with pupils from S1-6.
