# Supplementary figures and images for: EnLightenment: High resolution smartphone microscopy as an educational and public engagement platform
Source: Wellcome Open Res. 2018 May 3;2:107. Originally published 2017 Nov 6. [Version 2] doi: 10.12688/wellcomeopenres.12841.2 (PMC5861559; doi:10.12688/wellcomeopenres.12841.2)

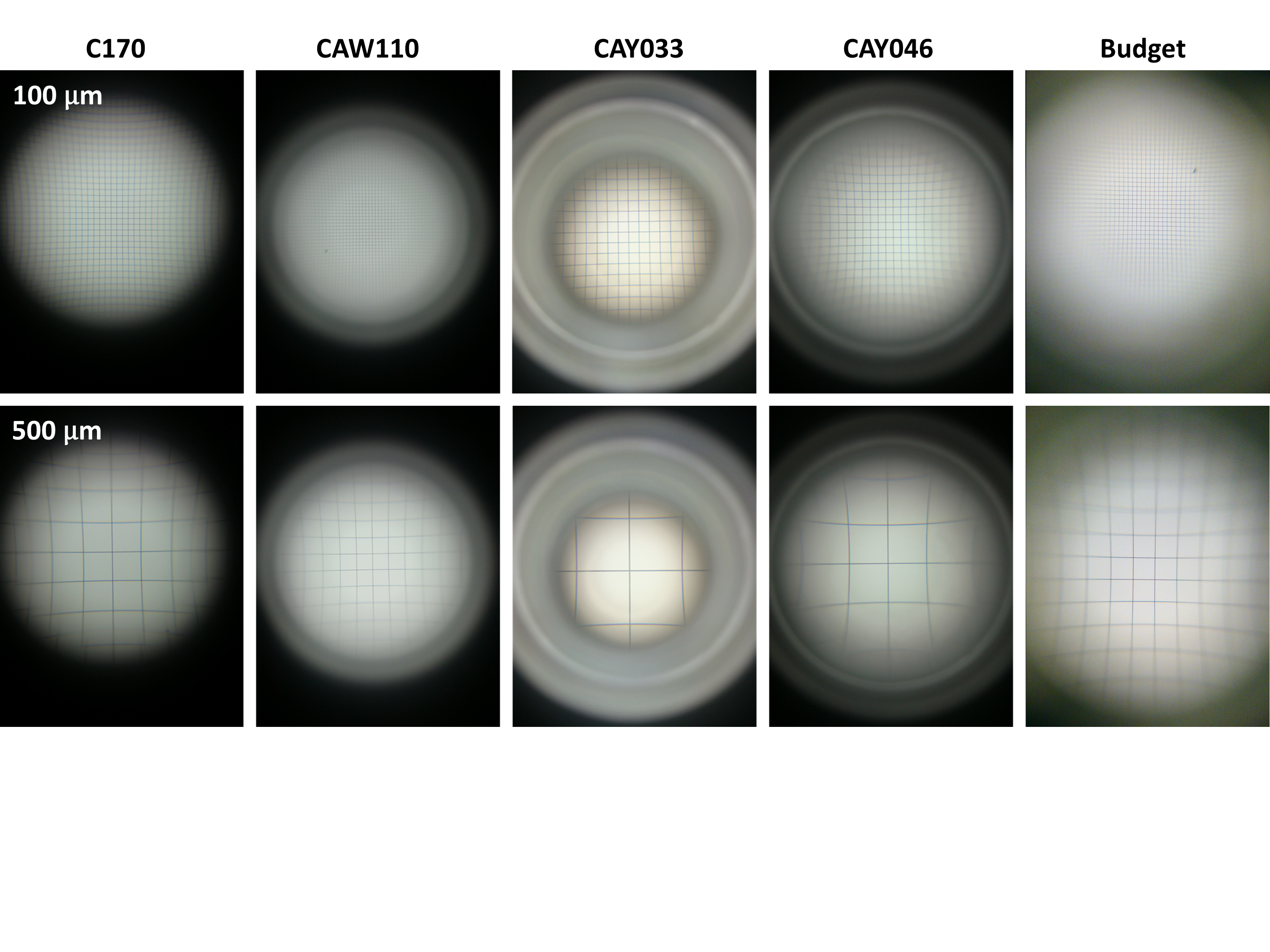

Supplement: Supplementary file 3 [file wellcomeopenres-2-14682-s0002.tgz › 789ee836-ec6a-4c26-a3f7-c8b6f5c5d8cd.tif]

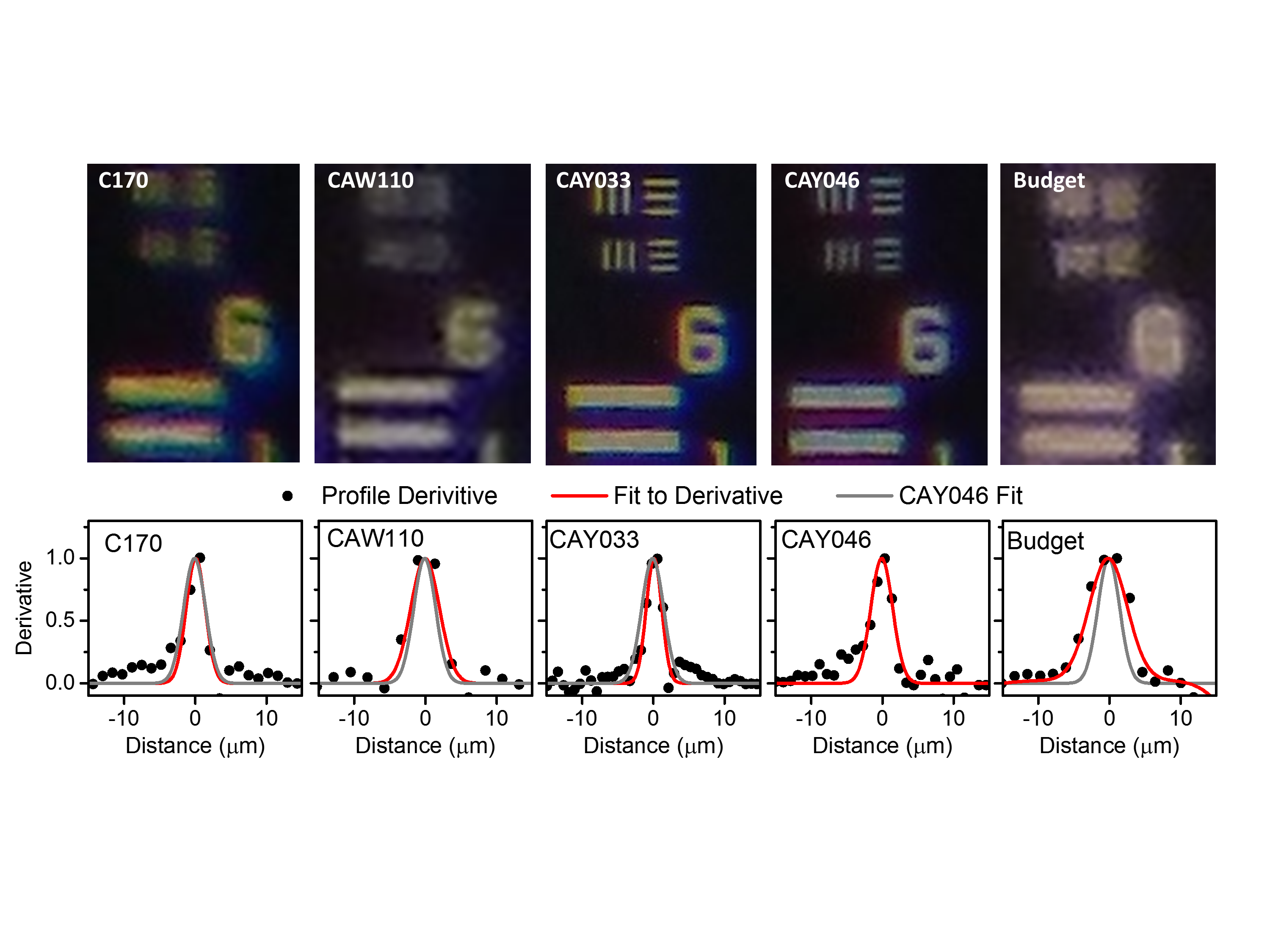

Supplement: Supplementary file 4 [file wellcomeopenres-2-14682-s0003.tgz › ed1b475e-78fd-402b-a5cf-23699de88ca9.tif]

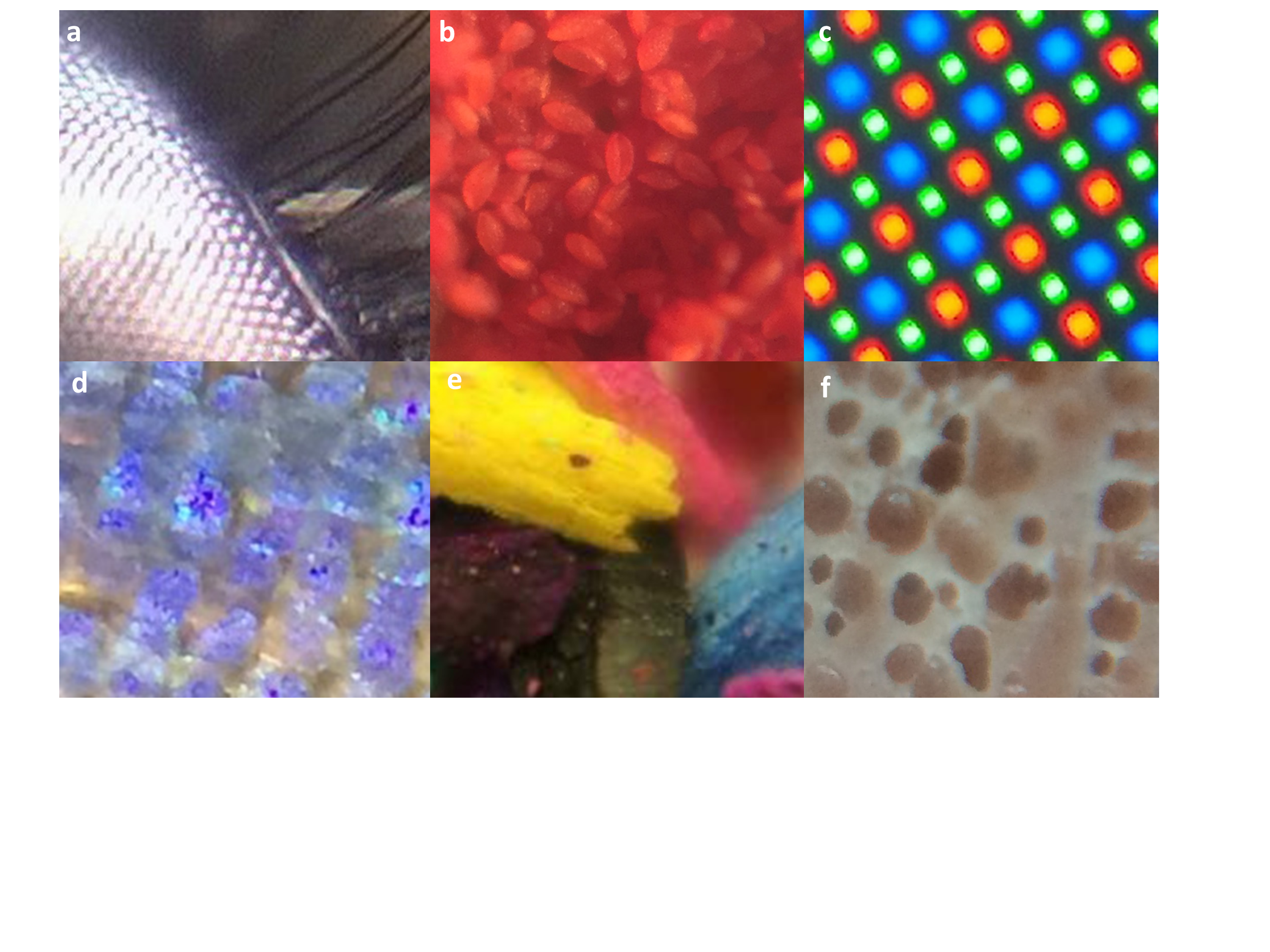

Supplement: Supplementary file 6 [file wellcomeopenres-2-14682-s0005.tgz › 565aafe6-201c-4427-8989-01249dbb9d0e.tif]

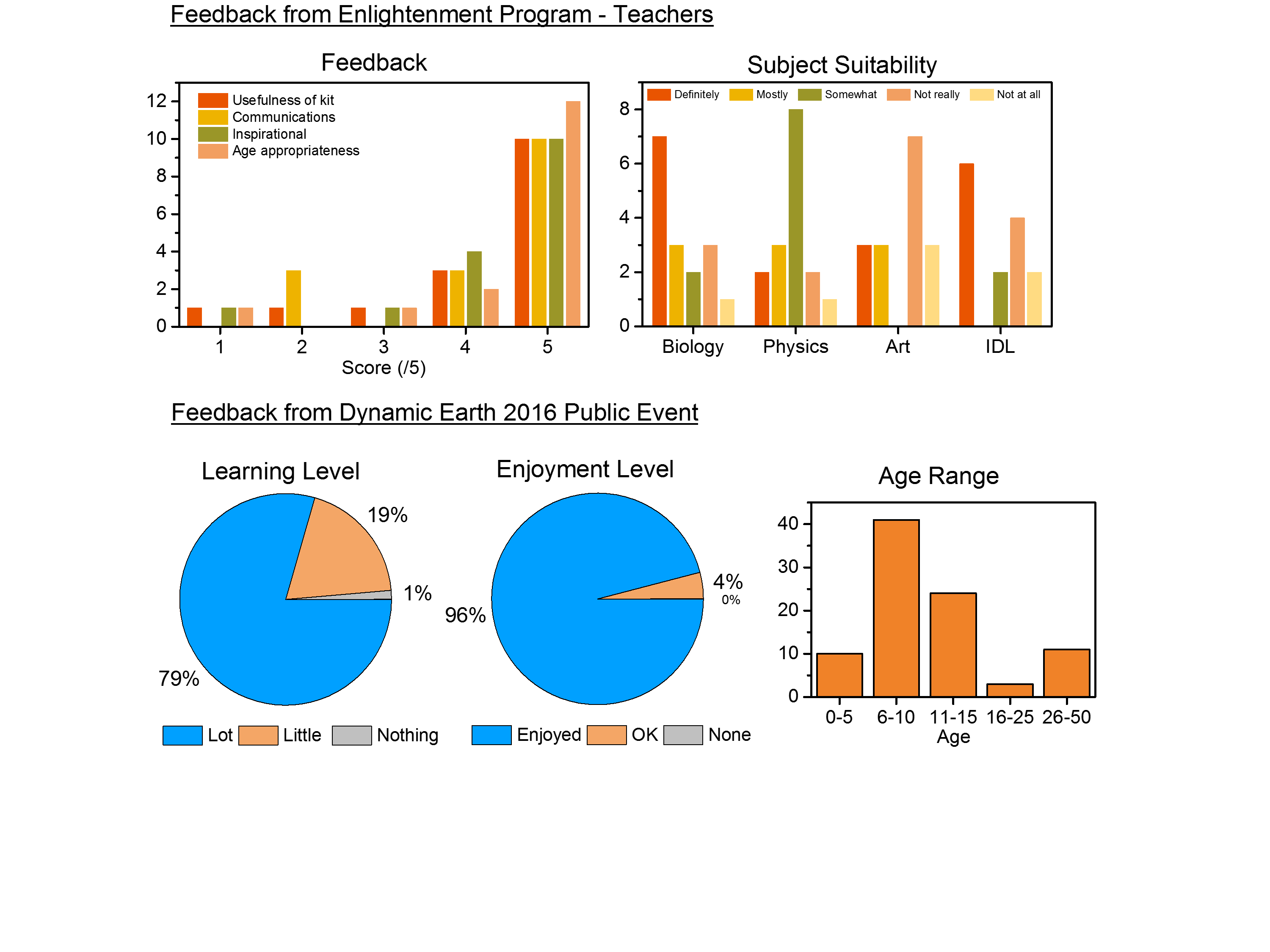

Supplement: Supplementary file 7 [file wellcomeopenres-2-14682-s0006.tgz › 81f43c19-df16-4cdd-9cee-5fb53fd86614.tif]
