# Supplementary material for: EnLightenment: High resolution smartphone microscopy as an educational and public engagement platform
Source: Wellcome Open Res. 2018 May 3;2:107. Originally published 2017 Nov 6. [Version 2] doi: 10.12688/wellcomeopenres.12841.2 (PMC5861559; doi:10.12688/wellcomeopenres.12841.2)
Supplement: Supplementary file 1 [file wellcomeopenres-2-14682-s0000.tgz › 6e4f6025-7904-4865-9672-f2ddec803db0.zip!/Supplementary File 1/Lesson Plan.pdf]

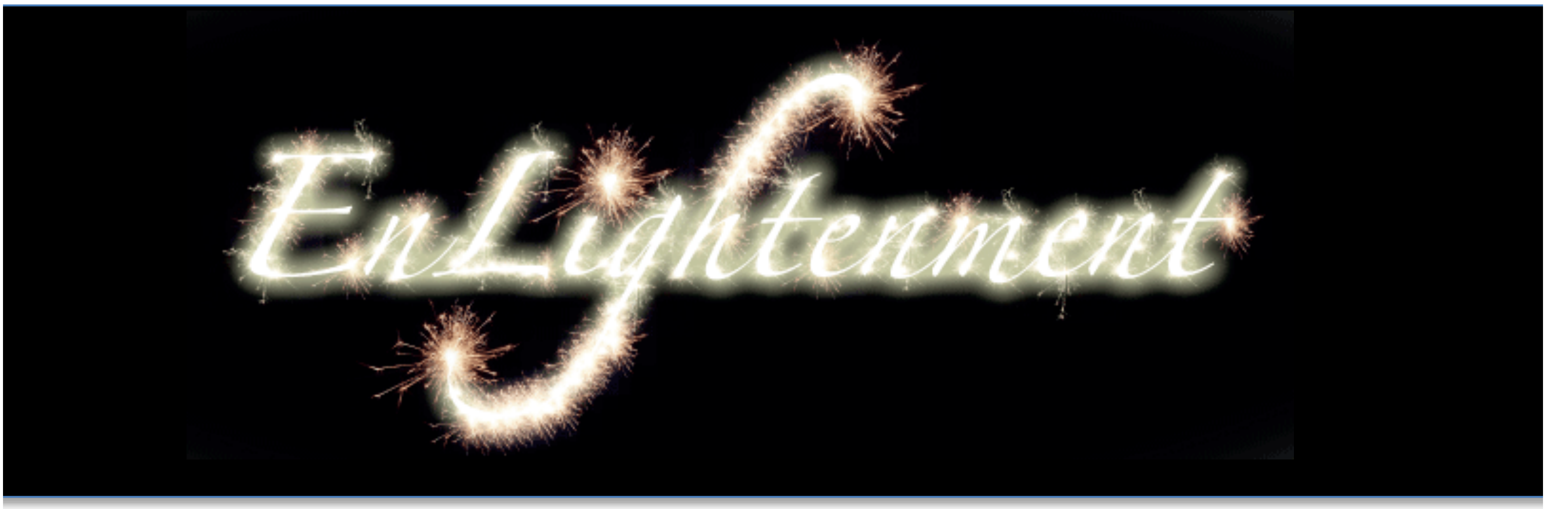

Light, physics, cells & hearts

E's & O's Covered:

1. **I have explored the structure and function of organs and organ systems and can relate this to the basic biological processes required to sustain life. SCN 3-12a**
2. **I have explored the role of technology in monitoring health and improving the quality of life. SCN 3-12b**
3. **Using a microscope, I have developed my understanding of the structure and variety of cells and of their functions. SCN 3-13a.**

Activity length - about 1 – 2 periods.

Materials:

1. **Unassembled Camera Phone Microscopes.**
2. **Pre-prepared microscope slides and business cards with text.**
3. **Daphnia samples set out in small beakers – 1 per group of 4. (Easier if beakers have plenty daphnia in them.)**
4. **1% alcohol solution in beakers – 1 per group of 4.**
5. **Energy Drink in beakers – 1 per group of 4.**
6. **Plastic droppers.**

1. Introduction to microscope parts and safe use.
2. Demo how to assemble microscope and how to view a business card printed with the word "and". Emphasise that the lens and the Perspex base both have a right and wrong way up – lens is fitted on the top side and the flat side of lens should be towards mobile phone. This can be checked as the image on phone will be blurred around the edge and only clear in the middle. Withhold lenses until end of assembly process so they are not lost.
3. Pupils build microscopes. Issue lenses and insert once finished. Offer business cards for imaging for pupils further ahead.
4. Demo how to collect daphnia in a dropper, place on slide and focus with microscope.
5. Collect daphnia, take pics, videos(slow-mo if possible). Count heart rates. Collate results and get a class average at the end.
6. Return daphnia to separate beaker – so they are not used again.

7. Repeat with a second daphnia and add rockstar and/or alcohol.
8. Record data into excel. Discuss any patterns, the structure and function of the heart, the influence of alcohol/caffeine on the body, the variety of structures in the daphnia.

Type of Solution	Heart Rate (BPM)					
	Concentration	Beats in 15s		Calculated BPM		Av. BPM
Water						
Caffeine						
Ethanol						
