# Supplementary material for: EnLightenment: High resolution smartphone microscopy as an educational and public engagement platform
Source: Wellcome Open Res. 2018 May 3;2:107. Originally published 2017 Nov 6. [Version 2] doi: 10.12688/wellcomeopenres.12841.2 (PMC5861559; doi:10.12688/wellcomeopenres.12841.2)

HERIOT WATT UNIVERSITY

EnLightenment

DIY SMARTPHONE MICROSCOPE CONTEST

TEACHER INSTRUCTIONS

Kit contents (x 5):

- 1 large acrylic plate
- 1 smaller acrylic plate (specimen plate)
- Box of bolts, nuts and washers
- Lens

Instructions (photo overleaf):

1. Remove white plastic covering from both acrylic plates (it's tricky!)
2. Use the bolts to add legs to the corners of the large acrylic plate to form the main part of the microscope. Make sure the logos are facing up.
3. Use washers and bolts to secure the legs. Add plastic end caps to act as feet (it may help to secure them on with a small piece of blu-tac)
4. Use a bolt to fix the specimen plate under the top plate, using the central countersunk hole. The specimen plate will hang loose at the opposite end, below the smaller drilled hole.
5. Secure the specimen plate in position using either a wingnut or nut - you may require washers between the plates to act as spacers, but this should be investigated by the pupils.
6. Add the smaller screw to the bottom end of the large acrylic sheet, inserting it only just through the drilled and tapped hole at that position.
7. Add the lens, placing it in the drilled hole next to the smaller screw. The lens sits on a small lip at the bottom of the hole and needs securing in place. Pupils should think about the best way to do this whilst making sure the lens isn't damaged or obstructed. A small drop of clear nail polish on the lip, or similar, works well. The lens has a best way up, pupils should investigate this before securing it in place by testing the microscope.
8. Find a light source – for example, natural light, the torch from the smartphone or a small LED lamp underneath the microscope. In the latter, you may need a diffuser to spread or limit the light - tissue paper work well here.
9. You can test the focus using white paper with simple writing – pen on paper or any printed media such as business cards or handouts are good first samples.
10. Finally, find some interesting samples and upload images to www.EnLightenment.hw.ac.uk to win prizes!

HERIOT WATT UNIVERSITY

EnLightenment

DIY SMARTPHONE MICROSCOPE CONTEST

HINTS & TIPS

- **There are more screws and bolts than needed and multiple ways to mount various parts of the microscope, this is to encourage pupils to think about the best possible design**
- **The acrylic top plate has a correct orientation; the bolts should be countersunk and flush on the top when complete. This is to allow for larger smartphones and tablets to be used.**
- **The lens also has an optimal way up, although it will work in both orientations. Pupils should study this but the results can be checked – when the lens is the wrong way up the image on the phone/tablet will be blurred around the edge and only clear in the middle.**
- **Withhold the lenses until the end of assembly process so they are not lost.**
- **The final images will be dark or blurry around the edges with only a smaller clear part in the centre. This is due to the lens but it gives much larger and clearer images in this central region (which you can zoom into on the phone/tablet).**
- **The specimen plate may bend over time, simply remove it and turn it around to counteract this effect.**
- **There is large scope for optimisation. Spacers between the plates may aid focussing.**
- **Make sure pupils enter their names and school when uploading to the website to be in with a chance of winning prizes!**

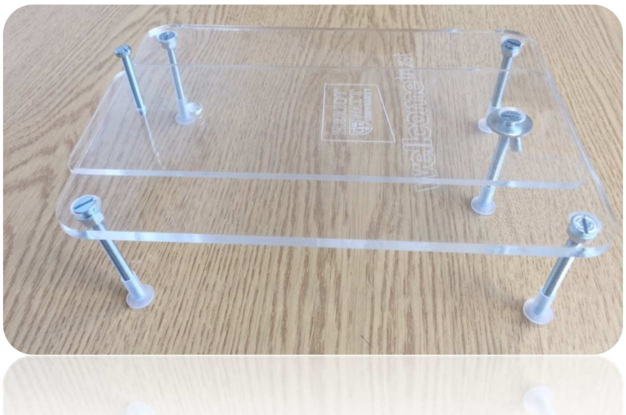

Supplement: Supplementary file 1 [file wellcomeopenres-2-14682-s0000.tgz › 6e4f6025-7904-4865-9672-f2ddec803db0.zip!/Supplementary File 1/Teacher Instructions.pdf]
