# Supplementary material for: EnLightenment: High resolution smartphone microscopy as an educational and public engagement platform
Source: Wellcome Open Res. 2018 May 3;2:107. Originally published 2017 Nov 6. [Version 2] doi: 10.12688/wellcomeopenres.12841.2 (PMC5861559; doi:10.12688/wellcomeopenres.12841.2)
Supplement: Peer review history

# Peer review: Referee response for version 1

**DOI:** 10.21956/wellcomeopenres.13914.r31688

**Published:** 2018-03-19

**Authors:** Young-Gu Ju

**Affiliations:** 1 Department of Physics, Kyungpook National University, Daegu, South Korea

I am very pleased to have a chance to review this paper. The paper deals with how to design and build a low cost smart-phone microscope for education and demonstrated the results of applying those developed toolkits to the students in many schools. Furthermore, the activity was well organized in terms of preparing teaching material, running a website to harvest the images from the students and giving feedback through awarding the winners.

 A smart-phone can be a very useful tool to draw attention of the students into a scientific activity if used properly. The activity shown in the paper can be a good example showing this kind of possibility. The collected images looks very good from artistic viewpoint. I hope this activity will lead to a little more advanced investigation into science for students in the future. For example, teaching image processing for quantitative analysis can be a good topic. For now, it seems a good start.

 As for the paper, I found several tiny mistakes, it would be good if they are corrected some way.

 1)

 page 5, right column, 7th line from the bottom :

 The focal lengths should be 1.8 - 6.0 mm instead of 18 - 60 mm. 

 (Typically, it is around 3 mm.)

 Otherwise, you cannot get the magnification mentioned in the paper.

 2)

 page 6, left column, line 3 : Digure 2b -> Figure 2b

 3)

 page 10, the last sentence

 (As for this, I am not sure because I am not good at English.)

 'complimentary to modern lifestyle and associated technology'

 -> 'complementary" to modern lifestyle and associated Technology'

I have read this submission. I believe that I have an appropriate level of expertise to confirm that it is of an acceptable scientific standard.

# Peer review: Referee response for version 1

**DOI:** 10.21956/wellcomeopenres.13914.r27640

**Published:** 2017-11-24

**Authors:** Mhairi Stewart

**Affiliations:** 1 University of St Andrews, St Andrews, UK

Firstly I would like to commend the authors on creating a simple and elegant instrument for general use and very importantly the understanding of microscopy. In particularly I feel the inclusion of a selection of building components in the kit to allow for interpretive builds is laudable, allowing particularly engaged participants to really explore microscope build, the limits of the apparatus, and expand their confidence and knowledge. The engagement activity was also well thought through and delivered, demonstrating the high value and impact of co-production and collaboration of engagement activities with stakeholders, in this case researchers, public engagement facilitators, teachers, and pupils.

 There are a very few points I would recommend be addressed and highlight them below.

 Learning objectives included imaging sub-cellular features. While the imaging of individual pollen grains was elegantly evident I wasn't aware of sub-cellular imagery in any of the submitted images. I would invite the authors to comment on this. If it is a simple limitation of use I don't believe it detracts at all from the smartphone microscope, however I feel it should be addressed in the text.

 The example teaching resources in the supplementary materials and on the website are clear instructions on use, data collection and logging. I felt however, that the teacher’s notes could have included more detail on the biological processes to support discussion. I understand of course this was not the core aim of this project, and may well have been addressed elsewhere, however in terms of teacher support and engagement it may be useful.

 I would have welcomed some further information regarding the evaluation for the public engagement activities. The teacher feedback form and data was well presented and clear to review. As one of the stated aims, I would like to see more comment on functionality and ease of use. This might be included in the ‘usefulness’ data return, however this is not clear.

 Of slight concern was interpretation of the inspirational qualities of the kit on the pupils from a teacher’s perspective. This is absolutely a useful measure however has to be interpreted with caution as a third person view of impact on another. I understand that individual participant evaluation in a remote delivery situation in order to collect baseline before and learning and inspiration after an activity is not appropriate or conducive to uptake. However, some information on this from the schools directly visited would be welcome.

 Individual evaluation from participants outside of schools was quoted, although from one source only. This did, however, reflect the learning and enjoyment levels of participants as does the number of entries received in the competition, so I am somewhat confident the assumptions made are correct, however I would suggest that inclusion of further data would confirm this and make the submitted paper stronger.

 Once again I do commend this activity and look forward to hearing how the work progresses from here.

I have read this submission. I believe that I have an appropriate level of expertise to confirm that it is of an acceptable scientific standard.

# Response

**Published:** 2017-12-14

**Authors:** Paul Dalgarno

**Affiliations:** Heriot Watt University, UK

Response to reviewer  – EnLightenment

We thank the reviewer for her encouraging and supportive remarks, and have addressed her suggestions below.

Referee Comment:

*Firstly I would like to commend the authors on creating a simple and elegant instrument for general use and very importantly the understanding of microscopy. In particularly I feel the inclusion of a selection of building components in the kit to allow for interpretive builds is laudable, allowing particularly engaged participants to really explore microscope build, the limits of the apparatus, and expand their confidence and knowledge. The engagement activity was also well thought through and delivered, demonstrating the high value and impact of co-production and collaboration of engagement activities with stakeholders, in this case researchers, public engagement facilitators, teachers, and pupils.*

*There are a very few points I would recommend be addressed and highlight them below.*

*Learning objectives included imaging sub-cellular features. While the imaging of individual pollen grains was elegantly evident I wasn't aware of sub-cellular imagery in any of the submitted images. I would invite the authors to comment on this. If it is a simple limitation of use I don't believe it detracts at all from the smartphone microscope, however I feel it should be addressed in the text.*

Response:

Thanks for this – we didn’t highlight these outcomes sufficiently in the text. Some of the entries from participating schools do indeed resolve intracellular structures. For example, in Supplementary Figure S3, that shows all 510 entered images, several show nuclei, plasma membranes and other organelles, with little sample preparation required to achieve this resolution. For example, nuclei and cell walls are clearly resolved in iodine stained onion skin cells ( http://enlightenment.hw.ac.uk/content/iodine-on-onion/).

We have modified the text to bring this out as follows;

“ **Submitted images**

The uploaded images demonstrate two core results. First, the diversity, inspiration and capabilities of 12–14 year olds who are engaged with science. Secondly, the potential and wide-ranging applications of a smartphone microscope engineered for simplicity and optical performance. Supplementary Figures S3a–d show collages of all uploaded images, and full size versions are accessible via the *EnLightenment* website. Subjects vary from insects and wildlife to plants, electronics, art, chemistry and engineering. Images were uploaded from a variety of devices covering most major tablet and smartphone manufacturers and models of the time period. Many submitted images clearly resolved sub-cellular structures. A prime example, resolving plant cell walls and nuclei, can be seen here; Iodine-on-onion submission. These examples show how sub-cellular features can be explored with the smartphone microscope in a standard classroom with minimal sample preparation.

Referee Comment:

*The example teaching resources in the supplementary materials and on the website are clear instructions on use, data collection and logging. I felt however, that the teacher’s notes could have included more detail on the biological processes to support discussion. I understand of course this was not the core aim of this project, and may well have been addressed elsewhere, however in terms of teacher support and engagement it may be useful.*

**

Response:

The teaching resources were developed in collaboration with high school teachers (including co-author Bryce), and we were advised to keep them to a minimum to allow teachers to interpret them in their own way, based on their pupils. The curriculum for excellence allows a degree of flexibility, and we were keen to create something to enable students to explore within a broad remit of understanding and learning. We also created the resource to be transferrable to all ages, not just S1-2, so chose not to align with specific deliverables for that area. Any future projects that may align more directly to specific parts of the curriculum would be co-developed with educators to be more specific as required. We are also investigating reaching more diverse audiences.

Referee Comment:

*I would have welcomed some further information regarding the evaluation for the public engagement activities. The teacher feedback form and data was well presented and clear to review. As one of the stated aims, I would like to see more comment on functionality and ease of use. This might be included in the ‘usefulness’ data return, however this is not clear.*

Response:

The most valuable feedback collected during the project was through conversations with teachers, and comments collated through the online surveys. We chose to focus on open ended questions, to allow us to modify kits in the future based on the feedback. In terms of functionality and ease of use, we asked for improvements from both teachers and pupils as felt these may differ. See below for further details.

Referee Comment:

*Of slight concern was interpretation of the inspirational qualities of the kit on the pupils from a teacher’s perspective. This is absolutely a useful measure however has to be interpreted with caution as a third person view of impact on another. I understand that individual participant evaluation in a remote delivery situation in order to collect baseline before and learning and inspiration after an activity is not appropriate or conducive to uptake. However, some information on this from the schools directly visited would be welcome.*

**

*Individual evaluation from participants outside of schools was quoted, although from one source only. This did, however, reflect the learning and enjoyment levels of participants as does the number of entries received in the competition, so I am somewhat confident the assumptions made are correct, however I would suggest that inclusion of further data would confirm this and make the submitted paper stronger.*

**

*Once again I do commend this activity and look forward to hearing how the work progresses from here.*

Response:

We absolutely agree with the referee on the challenges and importance of collecting meaningful evaluative data with these type of activities, and thus focused on having conversations with teachers both over the phone, and at events. This helped draw out valuable information about how we could improve the kits, which helped shape the next phase of the project. This feedback was collated for record in a teacher survey sent out at project end, the link to which is imbedded in the “feedback” section. Supplementary Figure S5 summarized the quantifiable aspects of this feedback. However, we have now published the full summary of all responses, which includes a large selection of written comments that helped shape future developments of the microscope, in Supplementary File 2.

We have correspondingly amended the final text in the opening paragraph of “Feebdback” section to read:

Supplementary File 2 shows a full collation of this feedback, including all comments received from the schools exactly as submitted via these forms. Although overwhelmingly positive, many valid points where raised for minor improvements. Some are non-viable due to cost or practicality (better lens’s, collapsible legs, more precise focussing etc), and some we intentionally avoided to enable pupil investigation and discovery (lens orientation, lens securing). However, we have already made modifications to the position of the objective lens for follow on projects and other small changes.
